# Fatigue, anxiety, depression and sleep quality in patients undergoing haemodialysis

**DOI:** 10.1186/s12882-021-02349-3

**Published:** 2021-04-28

**Authors:** Zakariya Al Naamani, Kevin Gormley, Helen Noble, Olinda Santin, Mohammed Al Maqbali

**Affiliations:** 1 School of Nursing and Midwifery, Queens’s University Belfast, Belfast, UK; 2 College of Nursing and Midwifery, Mohammed Bin Rashid University of Medicine and Health Science, Dubai, UAE; 3 Ministry of Health, Muscat, Oman

**Keywords:** Haemodialysis, Fatigue, Anxiety, Depression, Sleep quality, COVID-19

## Abstract

**Objective:**

Patients undergoing haemodialysis may experience troubling symptoms such as fatigue, anxiety, depression and sleep quality, which may affect their quality of life. The main objective of this study is to determine the prevalence of fatigue, anxiety, depression and sleep quality among patients receiving haemodialysis during the coronavirus disease 2019 (COVID-19) pandemic, and to explore the contributing predictors.

**Methods:**

A cross-sectional and descriptive correlational design using Qualtrics software was performed. Data were collected using the Functional Assessment of Cancer Therapy-Fatigue (FACT-F), the Hospital Anxiety and Depression Scale (HADS) and the Pittsburgh Sleep Quality Index (PSQI). Logistic regression analyses were used to explore the predictors that were associated with fatigue, anxiety, depression and sleep quality.

**Results:**

Of the 123 patients undergoing haemodialysis who participated, 53.7% (*n* = 66) reported fatigue, 43.9% (*n* = 54) reported anxiety, 33.3% (*n* = 41) reported depression and 56.9% (*n* = 70) reported poor sleep. Fatigue, anxiety and sleep quality (*P* < .05) were significantly associated with being female, and whether family members or relatives were suspected or confirmed with COVID-19. Logistic regression showed that being within the age group 31–40, having a secondary education level, anxiety, depression and sleep quality were the main predictors affecting the fatigue group.

**Conclusion:**

Fatigue, anxiety, depression and sleep quality are significant problems for patients receiving haemodialysis during the COVID-19 pandemic. Appropriate interventions to monitor and reduce fatigue, psychological problems and sleep quality amongst these patients are needed. This can help to strengthen preparations for responding to possible future outbreaks or pandemics of infectious diseases for patients receiving haemodialysis.

## Introduction

In 2020, the coronavirus disease 2019 (COVID-19) is considered a major challenge to healthcare systems worldwide. COVID-19 is a highly contagious virus that causes severe acute respiratory distress in humans. On March 2020, the World Health Organization (WHO) declared COVID-19 a global pandemic due to the rapid outbreak of the virus [1]. COVID-19 caused a significant and serious threat to people, especially those with underlying comorbidities such as patients with end-stage kidney disease (ESKD).

Patients with ESKD receiving in-centre haemodialysis are highly susceptible to COVID-19 infection because the haemodialysis environment is a high risk area during the virus outbreak and patients’ immunity is compromised due to the disease process [2]. These patients normally receive regular haemodialysis treatment, three times a week, in overcrowded and congested halls, mixed with various age groups from different backgrounds, making it difficult to adhere to all COVID-19 protection guidelines, especially the application of isolation and social distancing. Several studies have reported that haemodialysis patients have a significantly increased risk of transmission of infection with COVID-19 and a higher mortality rate compared with the general population [3, 4]. In a single center in Italy where 55 haemodialysis patients were infected with COVID-19, thirteen patients (52%) died [5].

Patients receiving haemodialysis experience debilitating psychological symptoms from the exhausting chronic haemodialysis treatment that negatively impacts on their mental health [6, 7]. The high prevalence and worrying consequences of COVID-19 might induce psychological distress among haemodialysis patients. Further understanding of the psychological disturbances that haemodialysis patients might experience during the COVID-19 pandemic is essential to promote good mental health. Therefore, this study is to determine the prevalence of fatigue, anxiety, depression and sleep quality among patients receiving haemodialysis during the coronavirus disease 2019 (COVID-19) pandemic, and to explore the contributing predictors.

## Method

### Study design

The study employed a large-scale cross-sectional, descriptive, correlational design. The survey was developed using an online platform (Qualtrics). Convenience sampling technique was used to collect responses. Participants were invited, through a link, to complete the questionnaire, which was sent by social media. Upon opening the link, participants were prompted to read the study’s introduction and decide whether they wanted to participate. Those who agreed to take part provided consent by clicking an “I consent to participate” box, presented prior to the start of the survey. This study adhered to the Strengthening the Reporting of Observational Studies in Epidemiology (STROBE) guideline for cross-sectional studies [8].

### Setting and sampling

The participants were recruited from across all Ministry of Health institutions in Oman. The study was performed from 1st September 2020 to 20th September 2020. The inclusion criteria for participating in the study were as follows: adult patient older than 18 years; diagnosed with ESKD and receiving haemodialysis for at least three months; no known psychiatric or neurological disorders that could interfere with study participation. Exclusion criteria included patients who were diagnosed with cancer or dementia.

### Measures

The questionnaires included detailed demographics, background history and scales, including the Functional Assessment of Cancer Therapy-Fatigue (FACT-F), the Pittsburgh Sleep Quality Index (PSQI), and the Hospital Anxiety and Depression Scale (HADS).

### Demographics

Information about participants’ age, sex, marital status, education and occupational status were obtained in the survey. In addition, participants were asked whether or not their relatives had either suspected or confirmed COVID-19?

### Fatigue

Fatigue was measured using the FACT-F This instrument consists of 13-items that assess self-reported fatigue over the past seven days [9]. Response options are on a 5-point Likert scale and range from 0 to 4. Total possible scores of the FACT-F range from 0 to 52. A higher score indicates less or no fatigue, whereas a lower score indicates more fatigue. Alexander et al. detected a cut-off point of equal or less than 36 indicating clinically significant fatigue [10]. The original FACT-F showed strong internal consistency (coefficient alpha 0.93–0.95) and good stability (test-retest r = 0.87) [9, 11].

### Depression and anxiety

The HADS includes 14 items assessing anxiety (7-items) and depression (7-items), which are rated on a 4-point Likert-type response (from 0 to 3) [12]. The scores in each subscale are computed by summing the corresponding items, with maximum scores of 21 for each subscale. The recommended cut-off values are ≥8 either for anxiety or depression [13]. The HADS showed very good internal consistency (Cronbach’s α = .83) [14].

### Sleep quality

The PSQI self-rated questionnaire assesses sleep quality over the past month [15]. The PSQI has 19-items that are categorised into seven components: subjective sleep quality, sleep latency, sleep duration, habitual sleep efficiency, sleep disturbances, use of sleep medications and daytime dysfunction. The score for each of the seven components can range from 0 to 3. The PSQI global score is calculated by the sum of the seven components, which ranges from 0 to 21, with a global score ≥ 5 indicating poor sleep quality in the previous month. The PSQI has acceptable reliability in Arabic (Cronbach’s α = .77) [16].

### Data analysis

The data was entered into the Statistical Package for Social Sciences (SPSS) version 25. In order to address the research questions, descriptive statistics were calculated in the form of means, standard deviations, standard errors, frequencies, percentages of all the scales and subscales and participant variables. Chi-squares (or Fisher’s exact test) were used to test whether the levels of fatigue, anxiety, depression and sleep quality differ in terms of demography and treatment. The correlation between fatigue, anxiety, depression and sleep quality was analysed used Pearson or Spearman’s rank correlation as appropriate. Logistic regression analyses were used to identify the predictive risk factors for fatigue, anxiety, depression and sleep quality, and the independent variable (age, sex, marital status, education, occupation and relatives having suspected or confirmed COVID-19. *P* > .05 was considered to be statistically significant for all analyses.

### Ethical considerations

Ethical permission was sought from the Research and Ethical Review and Approval Committee in the Directorate General of Planning and Studies at the Ministry of Health in Oman (MoH/CSR/18/9002). The confidentiality and privacy of the participants were maintained. The consent statement was obtained, as it is presented on the first screen of the survey tool. The study was performed according to the principles of the Declaration of Helsinki.

## Results

**Fig. 1:**
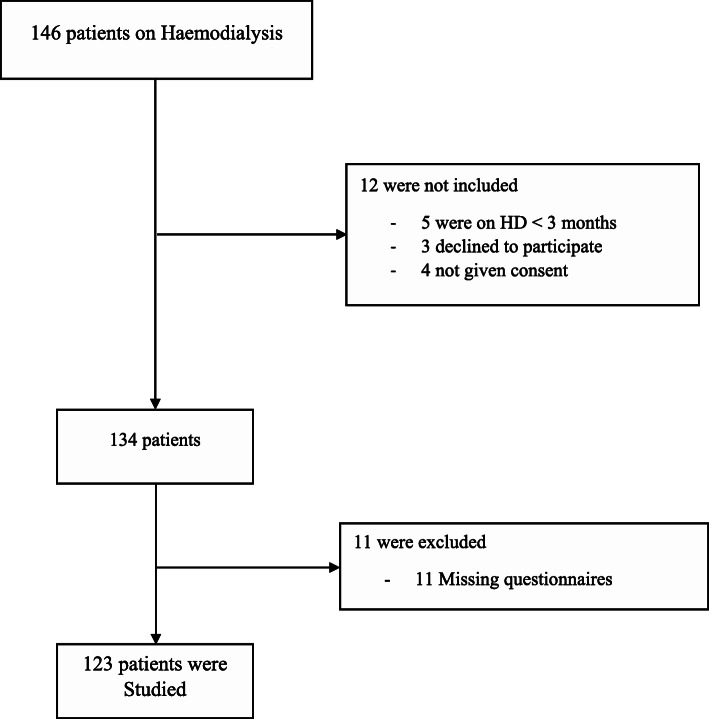
Flow diagram to show recruitment of participants

A total of 123 valid questionnaires were received through the online survey (Seen Fig. 1 for study flow diagram). The majority of the participants were male (67.3%, *n* = 83), and were married (82.1%, *n* = 101). The largest age group was those aged 31 to 40 years (43.1%, *n* = 53), followed by 41 to 50 years (30.1%, *n* = 37). Around half of the participants had confirmed or suspected COVID-19 among relatives (47.2%, *n* = 58). Overall, the prevalence of fatigue was 53.7%(*n* = 66), anxiety was 43.9% (*n* = 54), depression was 33.3% (*n* = 41), and poor sleep was 56.9% (*n* = 70). There were no differences in reporting fatigue, anxiety, depression and sleep quality according to age, marital status, educational level and occupation (P>0.05).

As shown in Table 1, there were significant differences in reports of fatigue according to gender (*p* < 0.05), with the highest proportion of fatigue among female participants (42.4% vs 21.1%) compared with the non-fatigue groups. There were significant differences in the reporting of fatigue (p < 0.05) according to those with relatives with suspected or confirmed COVID-19 (59.1% vs. 33.3%); they were more likely to have fatigue compared to the non-fatigue groups. Fatigue groups were more likely than non-fatigue groups to have anxiety (85.2% vs. 29%, *p* < .001), depression (80.5% vs. 40.2%, *p* < .001) and poor sleep quality (74.3% vs. 26.4%, *p* < .001).

**Table 1:** Demographic Characteristic of Participants (*N* = 123)

			Fatigued (n = 66, 53.7%)		Non-Fatigued (*n* = 57, 46.3%)			Anxiety (n = 54, 43.9%)		Non- Anxiety (*n* = 69, 65.1%)			Depression (n = 41, 33.3%)		Non-Depression (*n* = 82, 66.7%)			Poor Sleeper (n = 70, 56.9%)		Good Sleeper (n = 53, 43.1%)		
	n	%	n	%	n	%	*p*	n	%	n	%	*p*	n	%	n	%	*p*	n	%	n	%	*p*
**Gender**							.01					.00					.04					.01
Male	83	67.5	38	57.6	45	78.9		29	53.7	54	78.3		23	56.1	60	73.2		41	58.6	42	79.2	
Female	40	32.5	28	42.4	12	21.1		25	46.3	15	21.7		18	43.9	22	26.8		29	41.4	11	20.8	
**Age**							.06					.44					.89					.50
18–30	21	17.1	9	13.6	12	21.1		8	14.8	13	18.8		6	14.6	15	18.3		10	14.3	11	20.8	
31–40	53	43.1	34	51.5	19	33.3		26	48.1	27	39.1		17	41.5	36	43.9		34	48.6	19	35.8	
41–50	37	30.1	20	30.3	17	29.8		17	31.5	20	29		14	34.1	23	28		19	27.1	18	34	
More than 50	12	9.8	3	4.5	9	15.8		3	5.6	9	13		4	9.8	8	9.8		7	10	5	9.4	
**Marital Status**							.39					.52					.50					.16
Married	101	82.1	56	84.8	45	78.9		43	79.6	58	84.1		35	85.4	66	80.5		60	85.7	41	77.4	
Single	22	17.9	10	15.2	12	21.1		11	20.4	11	15.9		6	14.6	16	19.5		10	14.3	12	22.6	
**Education Level**							.30					.90					.16					.93
Basic	8	6.5	5	7.6	3	5.3		4	7.4	4	5.8		4	9.8	4	4.9		5	7.1	3	5.7	
Secondary	33	26.8	21	31.8	12	21.1		15	27.8	18	26.1		7	17.1	26	31.7		19	27.1	14	26.4	
Degree	82	66.7	40	60.6	42	73.7		35	64.8	47	68.1		30	73.2	52	63.4		46	65.7	36	67.9	
**Occupational**							.08					.24					.91					.06
Employed	87	70.7	44	66.7	43	75.4		37	68.5	50	72.5		28	68.3	59	72		44	62.9	43	81.1	
Unemployed	25	20.3	18	27.3	7	12.3		14	25.9	11	15.9		9	22	16	19.5		19	27.1	6	11.3	
Retired	11	8.9	4	6.1	7	12.3		3	5.6	8	11.6		4	9.8	7	8.5		7	10	4	7.5	
**Families or Relatives Suspected or Confirmed**							.00					.00					.52					.00
Yes	58	47.2	39	59.1	19	33.3		37	68.5	21	30.4		21	51.2	37	45.1		43	61.4	15	28.3	
No	65	52.8	27	40.9	38	66.7		17	31.5	48	69.6		20	48.8	45	54.9		27	38.6	38	71.7	
**Fatigue**												.00					.00					.00
No-Fatigue (FACIT-F ≥ 36)	57	46.3	–	–	–	–		8	14.8	49	71		8	19.5	49	59.8		18	25.7	39	73.6	
Fatigue (FACIT-F < 36)	66	53.7	–	–	–	–		46	85.2	20	29		33	80.5	33	40.2		52	74.3	14	26.4	
**HADS Anxiety**							.00										.00					.00
No Anxiety (HADS(A) < 8)	54	43.9	20	30.3	46	69.7		–	–	–	–		13	31.7	56	68.3		26	37.1	43	81.1	
Anxiety (HADS(A) ≥ 8)	69	65.1	49	86	8	14		–	–	–	–		28	68.3	26	31.7		44	62.9	10	18.9	
**HADS Depression**							.00					.00										.00
No Depression (HADS(D) < 8)	41	33.3	33	50	49	86		26	48.1	56	81.2		–	–	–	–		39	55.7	43	81.1	
Depression (HADS(D) ≥ 8)	82	66.7	33	50	8	14		28	51.9	23	18.8		–	–	–	–		31	44.3	10	18.9	
**PSQI**							.00					.00					.00					
Good Sleeper	53	43.1	14	21.2	39	68.4		10	18.5	43	62.3		10	24.4	43	52.4		–	–	–	–	
Poor Sleep	70	56.9	52	78.8	18	31.6		44	81.5	26	37.7		31	75.6	39	47.6		–	–	–	–	

Comparisons showed that depression groups were statistically significantly higher in female participants (43.1% vs. 26.8%) (*p* < 0.05). Depressed respondents were significantly more likely that non depressed respondents to have fatigue (50% vs. 14%, *p* < .001), anxiety (51.9% vs. 18.8%, *p* < .001) and poor sleep quality (44.3% vs. 18.9%, *p* < .001).

There were significant differences in reports of anxiety according to gender (female:46.3% vs. 21.7%) and those with relatives with suspected or confirmed COVID-19 (68.5% vs. 30.4%) (*p* < 0.05) compared with the non-anxiety groups. There were significant differences in the reporting of anxiety according to fatigue (86% vs. 14%, *p* < .001), depression (68.3% vs. 31.7%, *p* < .001), and poor sleep quality (62.9% vs. 18.9%, *p* < .001); all of whom were more likely to have anxiety compared to the non-anxiety groups.

The poor sleeper groups had significantly more female participants (41.4% vs. 20.8%) as did those with relatives with suspected or confirmed COVID-19 (61.4% vs. 28.3%) (*p* < 0.05) compared to the good sleeper groups. Poor sleep was more prevalent among participants with fatigue (78.8% vs. 31.6%, *p* < .001), anxiety (81.5% vs. 37.7%, p < .001) and depression (75.6% vs. 47.6%, p < .001), when compared with the good sleeper group.

### Predictive factors associated with fatigue, anxiety, depression and sleep quality

Four logistic regressions were conducted to identify predictors of fatigue, anxiety, depression and sleep quality (Table 2).

**Table 2:** Logistic regression analyses of factors associated with Fatigue, Depression, Anxiety, Sleep Disturbance Odds Ratio (95% CI)

	Fatigue		Depression		Anxiety		Poor Sleep	
	OR (95% CI)	*P*	OR (95% CI)	*P*	OR (95% CI)	*P*	OR (95% CI)	*P*
**Gender**								
Female	Ref							
Male	0.77 (0.22–2.76)	0.69	0.50 (0.16–1.53)	0.22	0.44 (0.13–1.56)	0.21	0.81 (0.24–2.68)	0.73
**Age**								
More than 50	Ref							
18–30	0.25 (0.01–4.5)	0.34	0.60 (0.05–6.78)	0.67	0.79 (0.06–10.62)	0.86	0.43 (0.05–4.00)	0.46
31–40	0.08 (0.01–1.07)	0.05	0.40 (0.05–3.43)	0.40	0.58 (0.06–5.56)	0.63	0.78 (0.12–5.16)	0.79
41–50	0.12 (0.01–1.49)	0.1	0.60 (0.08–4.40)	0.61	1.37 (0.16–12.12)	0.78	0.22 (0.04–1.34)	0.10
**Marital Status**								
Single	Ref							
Married	0.34 (0.06–1.78)	0.2	1.19 (0.30–4.77)	0.80	0.28 (0.06–1.25)	0.10	5.41 (1.12–26.28)	0.04
**Education Level**								
Degree	Ref							
Basic	0.38 (0.018–8.12)	0.53	0.72 (0.08–6.40)	0.77	2.01 (0.15–27.13)	0.60	0.11 (0.01–1.45)	0.09
Secondary	0.26 (0.07–0.99)	0.04	0.29 (0.08–1.01)	0.05	1.68 (0.47–5.95)	0.43	0.43 (0.12–1.53)	0.19
**Occupational**								
Employed	Ref							
Unemployed	0.23 (0.03–1.84)	0.16	0.66 (0.13–3.43)	0.61	0.39 (0.07–2.22)	0.29	13.36 (1.73–102.97)	0.01
Retired	1.24 (0.14–11.28)	0.84	1.83 (0.27–12.24)	0.53	0.35 (0.04–2.99)	0.34	3.95 (0.71–22.10)	0.12
**Families or Relatives Suspected or Confirmed**								
No	Ref							
Yes	0.67 (0.21–2.11)	0.49	0.43 (0.15–1.26)	0.12	4.15 (1.47–11.66)	0.01	2.52 (0.92–6.91)	0.07
Fatigue	–		4.93 (1.48–16.38)	0.01	7.37 (2.18–24.93)	0.00	3.53 (1.12–11.11)	0.03
Depression	7.98 (2.32–27.51)	0.00	–		0.38 (0.12–1.24)	0.11	0.57 (0.18–1.77)	0.33
Anxiety	6.35 (1.76–22.88)	0.00	0.37 (0.12–1.20)	0.1	–		0.27 (0.08–0.89)	0.03
Poor Sleep	3.86 (1.26–11.85)	0.01	0.57 (0.18–1.80)	0.34	0.30 (0.09–0.95)	0.04	–	

Five independent variables were significantly associated with fatigue (Table 2). The strongest predictor of fatigue was patients with depression were 7.9 times more likely to suffer fatigue (95% CI:2.32–27.51; p >.001). The second predicate of fatigue was anxiety; patients with anxiety were 6.3 times more likely to suffer fatigue (95% CI: 1.76–22.88; p >.001). Participants with poor sleep were 3.8 times (95% CI: 1.26–11.85; p >.001) more likely to experience fatigue. The 31–40 age group and secondary education level also significantly predicted fatigue (*p* < 0.05).

The logistic regression model of anxiety showed that patients with fatigue were 7.9 times more likely to have anxiety (95% CI: 2.18–24.93; p >.001). Patients with a family member with suspected or confirmed COVID-19 were 4.1 times more likely to have anxiety (95% CI: 1.47–11.66; p >.05). Poor sleep was significantly a predicator of anxiety (OR: .30; 95% CI: 0.09–0.95; p >.05).

In the depression logistic regression model, fatigue was the strongest predictor with participants being 4.9 time more likely to suffer depression (95% CI: 1.48–16.38; p >.05). The secondary education level also significantly predicted depression (OR: .29; 95% CI: 0.08–1.01; *p* = .05).

The unemployed patients had the strongest predicator and were 13.3 times more likely to report poor sleep (95% CI: 0.09–0.95; p >.05). Being married appeared to be the second predicate of poor sleep (OR 5.41; 95% CI: 1.12–26.28; *p* = .04). The models showed that patients having fatigue and anxiety were significant predictors of poor sleep.

## Discussion

To our knowledge, this is the first study to examine the prevalence of fatigue, anxiety, depression and sleep quality among patients undergoing haemodialysis in a middle eastern country during the COVID-19 pandemic. In this study, the prevalence of fatigue, anxiety, depression and sleep quality resulting from the pandemic among patients having haemodialysis is 53.7, 43.9, 33.3 and 56.9%, respectively. These results are higher than those found in previous research in patients receiving haemodialysis. Including fatigue (47%) [17], anxiety (17.5%) [18], depression (28%) [19] and sleep quality (48%) [20].

Compared to results of previous studies in general populations during the COVID-19 pandemic, the prevalence of anxiety, depression and sleep quality in this study was higher [21–23], which may be related to the high risk of dying of COVID-19 among patients with comorbidities and ESKD [24, 25]. This difference may be partially explained by the different isolation measures that were applied by countries to reduce the spread of COVID-19, which can affect patients receiving haemodialysis. In addition, the varied cultural norms, beliefs and values between countries may affect the status of fatigue, anxiety, depression and sleep quality. Another possible reason for the differences in prevalence the diversity of the assessment scale and healthcare system between the studies.

The present study indicates that female patients having haemodialysis had the highest levels of fatigue, anxiety, depression and sleep quality. These findings are consistent with general female populations during the COVID-19 pandemic [26–28]. Likewise, in this study, female patients were significantly associated with anxiety. Which may have been be due to worry about their family during the COVID-19 outbreak, and the consequences for their family if they were to become infected. Further, several studies have suggested that female gender is associated with increased prevalence of fatigue, anxiety, depression and sleep quality among patients receiving haemodialysis [29–31].

The results of this study show that participants have significantly increased risk of fatigue, anxiety and sleep quality if their family members are diagnosed with or have suspected COVID-19. This may be due to patients receiving haemodialysis being afraid of infection, either from the hospital setting or their families. It has been reported that haemodialysis patients who develop COVID-19 have high mortality rates [31, 32].

In this study, being unemployed appears to be the highest predicator of poor sleep. Previous research has reported similar findings [33, 34]. One explanation of this result is that unemployed patients do not have a regular routine in their daily lives. in the present study, depression was significantly associated with poor sleep. Likewise, several studies have found that depression has a significant effect on poor sleep quality in patients receiving haemodialysis [35, 36].

The study has a number of limitations. First, this study was conducted in Oman, which may limit generalization to other countries. Second the study utilised a cross-sectional design; therefore, it represents the evaluation of fatigue, anxiety, depression and poor sleep quality at one point in time, without longitudinal observation of participants. Finally, the study relied on the participants’ self-reporting questionnaires to assess psychological problems; however, this may differ from a clinical diagnostic interview. Further, objective measurement of sleep was not performed in the current study; however, the PSQI questionnaire showed good validity and reliability to measure sleep quality.

Altogether, fatigue, anxiety, depression and sleep quality are significant problems for patients undergoing haemodialysis. The results of the current study have a number of potential implications for interventions to improve psychological wellbeing of these patients. For example, organizations should provide counselling support services or online workshops and training material to enable patients to identify and overcome psychological problems. In addition, nurses play an essential role in helping to improve fatigue, anxiety, depression and sleep quality by providing high quality haemodialysis and creating a favourable environment for holistic care in renal dialysis units.

Further research should consider longitudinal design to identify the prevalence of fatigue, anxiety, depression and sleep quality before, during and after pandemic. Additionally, qualitative interview approaches will help to provide comprehensive in-depth understanding of fatigue, anxiety, depression and sleep quality and inform recommendations to improve further practice for those symptoms. Further research is needed to investigate the patients’ perceptions about the management strategy.

## Conclusion

This study is the first to describe fatigue, anxiety, depression and sleep quality among patients undergoing haemodialysis during the period of the COVID-19 outbreak. It will be helpful for dialysis staff and healthcare professionals as they identify risk predictors and the burdens of fatigue, anxiety, depression and sleep quality, and develop strategies to improve these symptoms among patient undergoing haemodialysis. Furthermore, it gives a solid foundation for further research, which should identify appropriate interventions to reduce fatigue, anxiety, depression and sleep quality of patients undergoing haemodialysis.

## Data Availability

The datasets generated and/or analysed during the current study are available from the corresponding author on reasonable request.
